# Menin: A Tumor Suppressor That Mediates Postsynaptic Receptor Expression and Synaptogenesis between Central Neurons of *Lymnaea stagnalis*

**DOI:** 10.1371/journal.pone.0111103

**Published:** 2014-10-27

**Authors:** Nichole Flynn, Angela Getz, Frank Visser, Tara A. Janes, Naweed I. Syed

**Affiliations:** Department of Cell Biology and Anatomy, and the Hotchkiss Brain Institute, Faculty of Medicine, University of Calgary, Calgary, Canada; University of Waterloo, Canada

## Abstract

Neurotrophic factors (NTFs) support neuronal survival, differentiation, and even synaptic plasticity both during development and throughout the life of an organism. However, their precise roles in central synapse formation remain unknown. Previously, we demonstrated that excitatory synapse formation in *Lymnaea stagnalis* requires a source of extrinsic NTFs and receptor tyrosine kinase (RTK) activation. Here we show that NTFs such as *Lymnaea* epidermal growth factor (L-EGF) act through RTKs to trigger a specific subset of intracellular signalling events in the postsynaptic neuron, which lead to the activation of the tumor suppressor menin, encoded by *Lymnaea MEN1* (*L-MEN1*) and the expression of excitatory nicotinic acetylcholine receptors (nAChRs). We provide direct evidence that the activation of the MAPK/ERK cascade is required for the expression of nAChRs, and subsequent synapse formation between pairs of neurons *in vitro*. Furthermore, we show that L-menin activation is sufficient for the expression of postsynaptic excitatory nAChRs and subsequent synapse formation in media devoid of NTFs. By extending our findings *in situ*, we reveal the necessity of EGFRs in mediating synapse formation between a single transplanted neuron and its intact presynaptic partner. Moreover, deficits in excitatory synapse formation following EGFR knock-down can be rescued by injecting synthetic *L-MEN1* mRNA in the intact central nervous system. Taken together, this study provides the first direct evidence that NTFs functioning via RTKs activate the *MEN1* gene, which appears sufficient to regulate synapse formation between central neurons. Our study also offers a novel developmental role for menin beyond tumour suppression in adult humans.

## Introduction

The neuromuscular junction (NMJ) has historically been used as the primary model to study cholinergic synapse formation. The steps underlying postsynaptic differentiation at the NMJ are well understood, and have been shown to involve presynaptically-derived agrin, which acts through muscle-specific kinase (MuSK) receptors to stabilize clusters of nicotinic acetylcholine receptors (nAChRs) at the postsynaptic membrane [1]. Much less is known however, about the mechanisms governing cholinergic assembly in the central nervous system (CNS). In light of growing evidence regarding the involvement of cholinergic neurons in cognition, learning, and memory, and the fact that cholinergic dysfunction underlies Alzheimer's and Niemann Pick disease C (NPC) [2], it is imperative to fully define the cellular and molecular mechanisms governing synaptogenesis between central cholinergic networks.

We have previously shown that excitatory synapse formation between pairs of individually identified *Lymnaea stagnalis* neurons requires an extrinsic source of NTFs (supplied by ganglion-conditioned media, or CM). Furthermore, NTF-mediated synapse formation relies upon the activation of receptor tyrosine kinases (RTKs), gene transcription and *de novo* protein synthesis [3]. More specifically, excitatory synapse formation between the presynaptic neuron visceral dorsal 4 (VD4) and the postsynaptic neuron left pedal dorsal 1 (LPeD1), relies upon activity-induced calcium influx in LPeD1, which in turn induces the expression of excitatory nAChRs. Without NTFs, the nAChR phenotype remains inhibitory by ‘default’ [4]. We have identified nAChR subunits in *Lymnaea* that are either cation- or chloride-selective [5], [6], however the NTF-triggered events that lead to the expression of the excitatory nAChRs and subsequent synapse formation remain unknown.

We previously identified a novel synaptogenic molecule, the *Lymnaea* homologue of the multiple endocrine neoplasia type 1 (*MEN1*) gene [7]. *Lymnaea MEN1* (*L-MEN1*) encodes the transcription factor menin, a tumor suppressor mutated in humans with MEN1 syndrome, a disorder characterized by tumors of the endocrine organs [8]. Menin is expressed in most tissues and some of the highest levels of *MEN1* mRNA are found in foetal brain tissue, suggesting that menin may also play a role in brain development [9]–[12]. Even though *MEN1* is so ubiquitously expressed and is conserved from *Drosophila* to humans [9], [11]–[16], its exact function remains unknown.

From the data presented in this study, we propose a novel mechanism by which RTK-mediated signalling governs cholinergic assembly both in cell culture and in the intact ganglion, by regulating the expression of menin in postsynaptic neurons. We further suggest that activation of the MAPK/ERK cascade may be an important intermediary step governing cholinergic synapse formation. In contrast to peripheral synapse formation at the NMJ, we demonstrate that the initial steps underlying postsynaptic differentiation in the CNS can all occur independently of presynaptic, activity-dependent mechanisms. This study is also the first to demonstrate the sufficiency of a single synaptogenic factor, menin, in rescuing synapse formation both *in vitro* and *in situ*, independent of neurite outgrowth.

## Materials and Methods

### Animals and cell culture

Freshwater snails (*Lymnaea stagnalis*) were raised in well-aerated aquaria at room temperature (20–22°) on a diet of romaine lettuce. Six-to-eight week old (10–15 mm shell length) animals were used for the isolation of individual neurons, whereas 2–4 month old snails (20–30 mm shell length) were used to make *Lymnaea* ganglion-conditioned media (CM). Due to the fact that *Lymnaea* are hermaphroditic, distinctions between male and female subjects do not apply.

The cell isolation protocol is well established and has been previously described in detail [17], [18]. Briefly, snails were de-shelled and anesthetised in a 10% solution of Listerine in normal *Lymnaea* saline (in mM: 51.3 NaCl, 1.7 KCl, 4.0 CaCl_2_, and 1.5 MgCl_2_) buffered to pH 7.9 with HEPES. The central ring ganglia (CRG - CNS of *Lymnaea*) were then removed under sterile conditions, and enzymatically treated with trypsin (2 mg/ml; T9201; Sigma-Aldrich) for 20–22 min followed by a trypsin inhibitor (2 mg/ml; T9003; Sigma-Aldrich) for 15 min. Both the trypsin and trypsin inhibitor were dissolved in defined medium (DM; L-15; Life Technologies; special order). The ganglia were then pinned down to a Sylgard dish in high osmolarity DM (750 µl of 1 M glucose to added 20 ml DM; raises normal osmolarity of the DM from 130–145 to 180–195 mOsm). After removing the outer and inner sheath protecting the ganglia, visually identified neurons were isolated by applying gentle suction through a fire-polished glass pipette that had been treated with Sigmacote. The neurons were then transferred to a poly-L-lysine-coated culture dish and plated in either DM (trophic factor-deficient media) or CM (trophic factor-rich media). For the drug-treated conditions, neurons were cultured overnight in DM+L-EGF (400 ng/ml), CM+PD153035 (EGFR inhibitor; 200 nM; 234490; EMD Millipore), CM+Nifedipine (L-type voltage-gated calcium channel inhibitor; 10 µM; 151743; ICN Biomedicals), CM+U0126 (MEK1/2 inhibitor; 40 µM; V1121; Promega), or the appropriate vehicle controls.

CM was prepared by incubating 12 central ring ganglia (previously washed in antibiotic saline containing 50 µg/ml gentamycin) in 6.5 ml of DM (serum-free 50% L-15 medium with added inorganic salts in mM: 40 NaCl, 1.7 KCl, 4.1 CaCl_2_, 1.5 MgCl_2_, and 20 µM gentamicin; buffered to pH 7.9 with HEPES) in Sigmacote-treated Petri dishes. The dishes were then placed in a humidified incubator at room temperature for 3 days to make 1X CM. The CM was frozen at −20°C if not used immediately. The ganglia were then re-washed several times in antibiotic saline, and incubated in fresh DM for another 4 days (2X CM). For this paper, 2X CM was used in all electrophysiological experiments and 1X CM for qPCR experiments.

### Generation of synthetic *L-MEN1* mRNA constructs

Whole CRG dissected from ∼25 snails were pooled and total RNA was isolated using the RNeasy lipid tissue mini kit (Qiagen) and reverse transcribed to cDNA using Superscript II reverse transcriptase (Life technologies) according to manufacturer's instructions. The *L-MEN1* cDNA containing the complete coding sequence (Accession no. AF395538) was PCR amplified with the primers (restriction sites underlined) *L-MEN1*-XhoI-5′ GATGATCTCGAGATGGCGGGCTTTCGAGACC and *L-MEN1*-NotI-3′ GATGATGCGGCCGCCTAGACTATTTCTCTCCTTGG using Q5 DNA polymerase (New England Biolabs) and subcloned into pBlueScript SK- (Clontech).

Synthetic poly A *L-MEN1* mRNA was synthesized with NotI-linearized pBluescript SK- (*L-MEN1*) as the template for *in vitro* transcription using T7 RNA polymerase from mMessage mMachine T7 Ultra kit (Ambion) according to manufacturer's instructions. After RNA synthesis, the DNA template was removed by performing a DNaseI digestion and the synthetic *L-MEN1* mRNA was purified using an RNeasy MinElute spin column (Qiagen). The quality and integrity of the RNA product was assessed by denaturing agarose gel electrophoresis.

### Single cell microinjections of mRNA

Single, postsynaptic LPeD1 neurons were isolated as described above, plated in DM, and left to adhere to the culture dishes for at least 15 min. The cells were then visualized with an inverted microscope (Axiovert 135; Zeiss) and with the aid of Narashige micromanipulators (Tokyo, Japan), impaled with an autoclaved glass microelectrode (1.5 mm internal diameter; World Precision Instruments) filled with 1 µl of synthetic *L-MEN1* mRNA (1.2–1.9 µg/µl). The mRNA was then microinjected into the cell using a PV800 pneumatic pump (10 pulses, 200–400 ms duration each, 4–6 psi; World Precision Instruments). The injected neurons were then left alone, or paired with a presynaptic neuron (VD4) overnight (16–18 hours). The next day, the cells were recorded from intracellularly or underwent cytosol extraction for qPCR.

### Electrophysiology

Conventional intracellular recording techniques were used to monitor single cell and synaptic physiology. Glass microelectrodes (1.5 mm internal diameter; World Precision Instruments) were pulled using a vertical pipette puller (Model 700C, David Kopf Instruments) and filled with a saturated solution of K_2_SO_4_. Only electrodes with resistances between 20–50 MΩ were used. Cells were visualized under an inverted microscope (Axiovert 135; Zeiss), and Narashige micromanipulators were used to impale the cells. The amplified electrical signals (Neuron Data Instrument) were relayed through a digitizer (Digidata 1440A; MDS) and recorded on Axoscope 10.2 (MDS).

To functionally test whether the nAChRs expressed on LPeD1 neurons were excitatory (appropriate – as seen *in vivo*) or inhibitory (inappropriate), microelectrodes were pulled as stated above. The tip was then enlarged under a microforge, to a size of <1 µm, and the microelectrode was filled with ACh (10^−6^ M dissolved in DM; A6625; Sigma Aldrich). The ACh-filled microelectrodes were then placed in close proximity to the cells, but at a distance to avoid mechanical stimulation (∼60 µm; determined using vehicle control pulses of DM only). ACh was then pressure applied onto the cell bodies (400 ms pulses; 10 psi) using a PV800 pneumatic pump, while recording from the cells intracellularly. To determine if excitatory or inhibitory receptors were functionally present, all cells were consistently held at a membrane potential between −55 and −60 mV (above the reversal potential for chloride). A response to a fixed pulse of ACh was deemed ‘excitatory’ if the cell depolarized, usually leading to a train of action potentials. The response was deemed ‘inhibitory’ instead, if the cell became hyperpolarized when held at −55 or −60 mV, or, if the cell was firing tonically prior to the pulse of ACh, a hyperpolarization and cessation of firing was induced. Furthermore, the response to ACh was seen to reverse at −70 mV in cells expressing inhibitory nAChRs, but did not in those expressing excitatory receptors.

To test synapse formation, pre- and postsynaptic neurons were impaled simultaneously. A synapse was confirmed to have formed if single action potentials induced in the presynaptic neuron, VD4, induced 1-for-1 excitatory postsynaptic potentials (EPSPs) or inhibitory postsynaptic potentials (IPSPs) in the postsynaptic neuron, LPeD1. At this stage, all cells were consistently held at a membrane potential of −100 mV, to accurately compare postsynaptic potential (PSP) amplitude in one culture condition versus another. To further characterize whether the synapses formed where excitatory or inhibitory, LPeD1 was brought up to a membrane potential of −60 mV (above the reversal potential for chloride). A train of action potentials was then induced in VD4, which either triggered a depolarization in LPeD1 (most often leading to spiking) in excitatory synapses, or a hyperpolarization and cessation of firing in LPeD1 in inhibitory synapses (a response which reversed at −70 mV, the reversal potential for chloride).

### qPCR

The single cell gene expression protocol was adapted from a method recently described in Nature Protocols [19], employing the Superscript III one-step reverse transcription polymerase chain reaction (RT-PCR) system with Platinum *Taq* DNA polymerase (12574-018; Invitrogen), ExoSAP-IT PCR product cleanup (78200; Affymetrix), and QuantiTect SYBR green PCR kit (204145; Qiagen) systems according to manufacturers' instructions. First, the culture media was removed and replaced with sterile saline via perfusion with a peristaltic pump (MINIPULS2; Gilson). Neuronal cytoplasm was then isolated by applying suction through a sterilized patch electrode (1.5 mm internal diameter; 603000; A-M Systems), leaving the nucleus behind in the culture dish. Because the *Lymnaea* genome has yet to be fully sequenced, intron spanning quantitative polymerase chain reaction (qPCR) primers could not be designed to discriminate between genomic DNA and intron-less cDNA (thereby guaranteeing that any product amplified from genomic DNA would be much larger than that amplified from the desired cDNA). Isolating the cytoplasm from the nucleus circumvented this problem, ensuring that the qPCR signal was derived exclusively from mRNA and not contaminated by the presence of genomic DNA. Cytosolic samples from single neurons were then deposited into 5 µL RT-PCR reaction buffer, frozen immediately on dry ice, and transferred to storage at −80°C. RT-PCR was performed using gene specific primers and 35 amplification cycles using an Eppendorf Mastercycler nexus GSX1 (Table 1). Because of the minute quantity of starting material, this initial amplification was necessary for signal detection during the qPCR reaction and did not impact the quantification of relative changes in gene expression. Single cell samples were then pooled and stored at −20°C until relative expression of the genes of interest (GOIs) were determined via qPCR using an Eppendorf Mastercycler ep realplex and primers directed against a region of approximately 80–120 bases (Table 2). Three negative controls were put in place to determine that the qPCR signal was specific to mRNAs pertaining to the GOIs: (1) external solution samples were collected and subjected to qPCR, (2) LPeD1 cytoplasm samples were analysed via qPCR without reverse transcription, and (3) no template controls using only the qPCR primers. All conditions were found to be negative (not shown). Efficiency values were determined for each primer set and ranged between 93–106% (Table 2), and R^2^ values ranged between 0.92–0.99. The qPCR products were sequenced to verify the correct identity of the amplified product.

**Table 1:** RT-PCR Gene Specific Primers (GSPs).

Target	Accession Number	5′ GSP Sequence	3′ GSP Sequence
*L-MEN1*	AF395538.1	TCGAGACCGAGCGAAGAAAC	TTTCGTGCAGATCCTGTTGG
β tubulin	X15542.1	TCCTACTTTGTGGAATGGATCC	ATGACGAGAATTATGTCATTAGAC
18s rRNA	Z73984.1	CTGGTTGATCCTGCCAGTAG	CTTCCGCAGGTTCACCTAC

Gene specific primers used for RT-PCR performed on cytosol samples isolated from single LPeD1 neurons.

**Table 2:** qPCR Gene Specific Primers (GSPs).

Target	5′ Primer Sequence	3′ Primer sequence	Efficiency
*L-MEN1*	TGGAGTTCGCTGTCTCGAAG	CAAAGGCAACACCAAAGCAA	93.52
β tubulin	ATCCAGGAGCTCTTCAAGCG	CTGTGAACTCCATCTCGTCC	105.8
18s rRNA	CACGGGGAGGTAGTGACG	GCCCTCCAATGGGTCCTC	103.4

Gene specific primers used for single cell qPCR. The relative expression of *L-MEN1* and two reference genes (β tublin and 18s rRNA) was determined via qPCR using primers directed against a region of ∼80–120 bases. Efficiency values were determined for each primer set.

### *In situ* transplantation of single LPeD1 neurons

To study synapse formation *in situ*, *Lymnaea* CRG were isolated and pinned to Sylgard dishes as described above. In half of the CRG, individual LPeD1 neurons were ablated by pronase injection (5% in *Lymnaea* saline; 537011; Molecular Probes). Specifically, the pronase solution was loaded into a glass microelectrode (Sigmacote-treated; 1 µm tip diameter; 1.5 mm internal diameter; World Precision Instruments), mixed with a 5% solution of the dye Lucifer yellow (LY), and injected in LPeD1 via pressure applied through a Gilmont microsyringe under the aid of a dissection microscope (Stemi SV 6; Zeiss; Germany). The cell bodies were completely fragmented within 1 hr. Healthy LPeD1 cells were isolated from the remaining CRG, and plated in DM in hemolymph-coated dishes (to prevent the cells from adhering to the dish) where they awaited transplantation. Once cell ablation was complete, individual LPeD1 neurons were transplanted into the pedal ganglia, in the same location where the host LPeD1 would have been previously found. Synapse formation was tested via intracellular recording 48–72 hours following transplantation.

A subset of healthy neurons were injected with LY prior to isolation [20] to determine normal morphology, whereas most transplanted neurons were filled with the dye 24–72 hours following transplantation. Briefly, the tips of glass microelectrodes (1.5 mm internal diameter; World Precision Instruments) were filled with a saturated solution of LY, and backfilled with LiCl_2_ (0.4 M in deionized H_2_O). With the aid of micromanipulators (MPC 200; Sutter Instrument Co.; USA), the cells were then impaled under the visual guidance of an upright stereoscope (model M5A; Wild Leitz; Switzerland). Prior to impalement, a constant holding current of +20 pA was applied to the electrodes to prevent dye leakage. Upon penetration, this current was switched to −20 pA to deliver the LY dye into the cell body. Approximately once every minute the negative current was turned off momentarily to allow LPeD1 to fire several action potentials, which aided the spread of the dye along the axon. Dye injection itself was performed in a darkened room with no direct light source to prevent bleaching. Cells were sufficiently loaded with LY dye after approximately 10 min, and the CRG were stored in *Lymnaea* saline overnight at 4°C. The following day, CRG were fixed in 4% PFA in 1× PBS for 3 hours at RT. After fixation, the CRG were dehydrated in a series of 30 min ethanol (EtOH) incubations (2 incubations in each concentration of 50%, 70%, 90%, and absolute EtOH). The ganglia were then de-fatted in DMSO (30 min) followed by clearing with methyl salicylate (10 min), and mounted on glass slides in methyl salicylate. The CRG were imaged with a fluorescent microscope (Axioskop; Zeiss; Germany) fitted with a digital camera (Canon Powershot G9), under 4× and 10× objective lenses. Excitation of LY dye was achieved using a blue-violet filter (395–440 nM) and the resulting fluorescence (∼542 nM) collected using a chromatic beam splitter (FT 460) and barrier filter (LP 470). All filters were acquired from Zeiss (Germany).

### Antisense knockdown of the L-EGFR in LPeD1 neurons prior to single cell transplantation

LPeD1 neurons were isolated and plated in DM in hemolymph-coated culture dishes. Control cells were left untreated, while two other groups of cells were incubated in either L-EGFR dsRNA or *Lymnaea* acetylcholine-binding protein (L-AChBP) dsRNA (negative control; glial protein). The 580 bp L-EGFR dsRNA and the 450 bp L-AChBP dsRNA were prepared and demonstrated to be effective previously [7], [21]. The dsRNAs were added to the culture medium at a concentration of 200 ng/ml for 24 hr prior to transplantation. Synapse formation was tested 48–72 hours following transplantation via intracellular recordings.

### Rescue of synapse formation in situ by injection of synthetic *L-MEN1* mRNA prior to single cell transplantation

Isolated CRG were incubated for 4–6 hours in wells containing either: (1) DM, (2) DM+ L-EGFR dsRNA, or (3) DM+L-AChBP dsRNA. In a subset of the CRG incubated in DM+L-EGFR dsRNA, LPeD1 neurons were injected with synthetic *L-MEN1* mRNA or molecular grade water as described above. 2–3 hours post-injection, LPeD1 neurons were isolated and transplanted into host CRG, in which the native LPeD1s were previously ablated by pronase injection. Synapse formation was tested 48–72 hours later following transplantation via intracellular recordings.

### Statistical Analysis

The percentages of cells that expressed excitatory nAChRs, and the percentage of cell pairs that formed synapses were compared using Pearson's Chi-squared test. If a general effect was discovered with an omnibus Chi-squared test (*P*<0.05), individual treatments were compared to one another with *post hoc* Chi-squared tests to analyze significance. Synaptic strength between VD4-LPeD1 neurons formed *in vitro* was analyzed using an independent samples t-test. Synaptic strength between VD4-LPeD1 neurons formed *in situ* was compared using a univariate analysis of variance (ANOVA), with the culture treatment as the fixed factor and the mean excitatory postsynaptic potential (EPSP) amplitude as the dependent variable. *Post hoc* multiple comparisons were performed using Tukey's test. All of the above statistical analyses were performed using SPSS Statistics 21 for Windows. Calculations determining relative gene expression, normalized to the expression of the reference genes β-tubulin and 18 s rRNA, and significant differences (via pair wise fixed reallocation randomisation test) were determined using the relative expression software tool (REST) [22].

## Results

### CM-derived NTFS and L-EGF are sufficient for the expression of excitatory nAChRs

We have previously shown that CM serves as an extrinsic source of NTFs required for the postsynaptic expression of excitatory nAChRs in *Lymnaea* neurons. In the absence of CM, the postsynaptic neuron LPeD1 expresses inhibitory nAChRs by ‘default’, and as a consequence, proper excitatory synapses do not form [4]. The precise identity of the NTFs in CM that are responsible for inducing the expression of excitatory nAChRs however, has not been fully determined.

In 2000, Hermann et al. identified and cloned *Lymnaea* EGF (L-EGF) from *Lymnaea* albumen glands, the first EGF homologue to be discovered in an invertebrate with neurotrophic actions [23]. While the source of L-EGF in the CNS is currently unknown, it is secreted rather than membrane-bound, and could act at long distances as a circulating hormone produced in the albumen gland. It has however, been shown to be highly expressed in juvenile CRG at a time when circuits are developing, so it may also be produced locally in the CNS during synaptogenesis [23]. In 2008, we demonstrated that L-EGF signals through an ErbB-type receptor, the *Lymnaea* EGFR (L-EGFR). This receptor, which has also been cloned, is required for excitatory synapse formation between VD4 and LPeD1 [7]; however it is unknown whether these effects involve pre- or postsynaptic sites. We thus opted to use single cells to investigate our hypothesis that L-EGF is sufficient to induce the expression of excitatory nAChRs in postsynaptic LPeD1 neurons.

**Figure 1:**
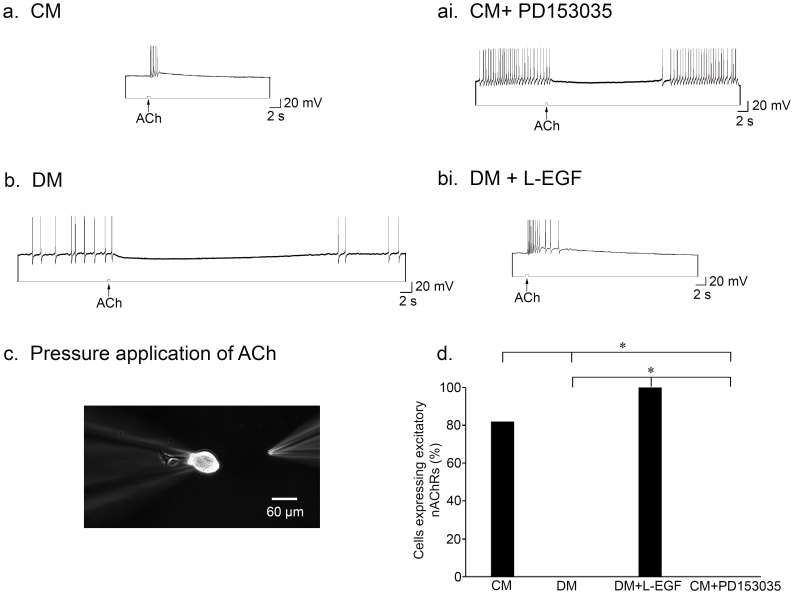
L-EGF is sufficient to induce the functional expression of excitatory nAChRs. (**a**) Single LPeD1 neurons were cultured in CM and impaled with intracellular electrodes. Exogenous application of ACh (10^−6^ M) triggered an excitatory response in the postsynaptic neuron, LPeD1 (n = 33). (**ai**) The EGFR inhibitor PD153035 (200 nM) prevented the CM-induced expression of excitatory nAChRs (n = 16). (**b**) Single LPeD1 neurons cultured in the absence of trophic factors (DM) exhibited an inappropriate (does not exist *in vivo*), inhibitory response to exogenous ACh application by ‘default’ (n = 9). (**bi**) L-EGF (400 ng/ml) was sufficient to rescue the expression of excitatory nAChRs in LPeD1 neurons cultured in DM (n = 5). (**c**) Experimental preparation. Phase contrast image of a single LPeD1 neuron impaled by a sharp intracellular electrode at 10× magnification. Another electrode with a tip diameter of 1–5 µm was used to pressure apply ACh onto the cell body of LPeD1, at a distance that was sufficient not to cause a mechanical stimulation artifact. (**d**) Summary of the percentage of cells that functionally exhibited excitatory nAChRs with a significance of *P*<0.001, as determined using Pearson's Chi-squared test.

To test the above hypothesis, we plated single LPeD1 neurons in CM, DM, or DM+L-EGF (400 ng/ml) overnight, for 16–18 hours. The next day, we recorded intracellularly from these neurons, while pressure-applying (“puffing”) ACh (10^−6^ M) onto their cell bodies (Fig. 1 c). In CM, 82% of the neurons expressed excitatory nAChRs (as normally seen *in vivo*) (n = 33; Fig. 1 a). On the other hand, none of the cells plated in the absence of NTFs (DM) expressed excitatory receptors (n = 9; Fig. 1 b). Excitatory nAChRs were however expressed in 100% of cases, when DM was supplemented with L-EGF (n = 5; Fig. 1 bi). We thus confirm that CM is required for the expression of appropriate, excitatory nAChRs (χ^2^ (1) = 20.618, *P*<0.001), and further demonstrate that L-EGF is sufficient to induce the expression of excitatory receptors in the absence of CM (χ^2^ (1) = 14.000, *P*<0.001) (Fig. 1 d). Although L-EGF functionally mimics the effects of CM (χ^2^ (1) = 1.080, *P* = 0.299), we opted to use CM for the rest of our experiments, as we feel that it best represents the *in vivo* condition by providing a full complement of NTFs that would be present in intact CRG.

### EGFRs are required for the expression of excitatory nAChRs

We have shown previously that the EGFR receptor inhibitor PD153035 blocks both CM and L-EGF-induced formation of excitatory synapses *in vitro*, as does L-EGFR knockdown with double-stranded RNA (dsRNA) [7]. We hypothesize that EGFRs act postsynaptically, and propose that the L-EGFR is necessary for the expression of excitatory nAChRs in LPeD1 neurons. To determine if functional excitatory receptors were present, we plated single LPeD1 neurons in CM or CM+PD153035 (EGFR inhibitor, 200 nM) overnight. The next day, we monitored the activity of the cells through intracellular recording, while simultaneously puffing 10^−6^ M ACh onto their somata. We found that 82% of the neurons cultured in CM expressed excitatory nAChRs (n = 33; Fig. 1 a). In contrast, none of the neurons cultured in CM+PD153035 exhibited an excitatory response to ACh (n = 16; Fig. 1 ai). As such, we conclude EGFR activation is required for the functional expression of excitatory nAChRs in postsynaptic, LPeD1 neurons (χ^2^ (1) = 29.157, *P*<0.001) (Fig. 1 d).

### Calcium influx through L-type voltage-gated calcium channels is required for the expression of excitatory nAChRs

Calcium is an important second messenger that induces signal transduction and the eventual regulation of the transcription of hundreds of genes to promote neurite growth, synapse formation, and synaptic plasticity [24]. That being said, the mode of calcium entry can determine whether or not gene expression will likely take place [25]–[27]. Previously, we demonstrated that the NTFs present in CM induce a rise in activity in postsynaptic (LPeD1) but not presynaptic (VD4) neurons, leading to calcium oscillations mediated predominantly by L-type voltage-gated calcium channels (VGCCs). This rise in postsynaptic activity, is necessary for the expression of excitatory nAChRs [4]. Therefore, we hypothesize that L-type VGCCs are also required for the expression of nAChRs.

**Figure 2:**
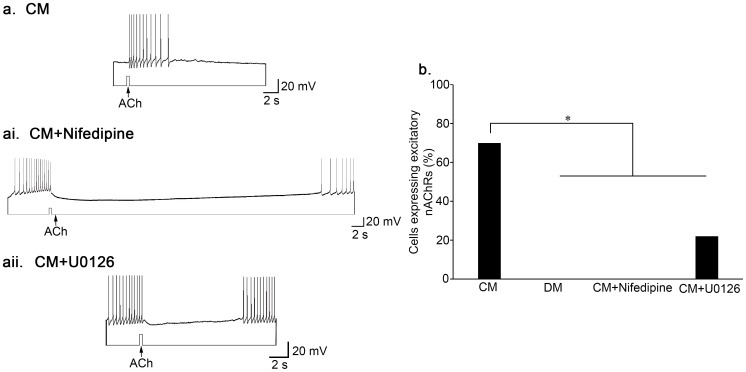
Calcium influx through L-type VGCCs and MAPK/ERK are required for the expression of excitatory nAChRs. (**a**) Single LPeD1 neurons were cultured in CM and impaled with intracellular electrodes. Exogenous application of ACh (10^−6^ M) triggered an excitatory response in the postsynaptic neuron, LPeD1 (n = 30). (**ai**) Exogenous application of ACh to LPeD1 somata induced an inhibitory response in the presence the L-type VGCC blocker nifedipine (n = 9) (10 µM). (**aii**) The expression of excitatory nAChRs was also prevented by the MEK1/2 inhibitor, U0126 (40 µM) n = 9. (**b**) Summary of the percentage of cells that functionally exhibited excitatory nAChRs with a significance of *P*<0.05, as determined using Pearson's Chi-squared test.

To investigate the functional expression of nAChRs, we plated single LPeD1 neurons in CM or CM+nifedipine (L-type VGCC antagonist, 10 µM) overnight. The next day, we monitored the activity of the cells through intracellular recording, while simultaneously puffing 10^−6^ M ACh onto their somata. In the presence of CM, 70% of cells expressed excitatory nAChRs (n = 30; Fig. 2 a). When nifedipine was present however, none of the cells expressed excitatory nAChRs (n = 9; Fig. 2 ai). L-type VGCCs are thus required for CM-induced, excitatory nAChR expression in single LPeD1 neurons (χ^2^ (1) = 13.650, *P*<0.001) (Fig. 2 b).

### MAPK/ERK activity is required for excitatory nAChR expression

The MAPK/ERK cascade is the prototypical cascade in the MAPK family. ERK1 and ERK2, the output of the cascade, are phosphorylated by MEK1 and MEK2, and MEK1/2 are activated upstream by Raf-1, and B-Raf [28]. The entire cascade is set in motion by extracellular signalling events, such as NTF stimulation, which recruit and activate the G-proteins Ras or RAP-1 via RTKs, which then activate the Rafs [29]. While we now know that ERK is required for several types of synaptic plasticity as well as learning and memory [30], [31], we have yet to determine its involvement in synapse formation.

We predicted that activation of ERK1/2 is required for CM-induced excitatory nAChR expression in LPeD1 neurons. To test this, we plated single LPeD1 neurons in CM+DMSO (0.4%; vehicle control), DM, or CM+U0126 overnight (40 µM). The MEK inhibitor U0126 was chosen as it is more potent than its counterpart, PD98059. Both agents non-competitively target MEK, and therefore MEK's only substrate ERK, and are more specific than other kinase inhibitors because they do not compete with ATP binding [28]. While recording intracellularly, we found that 70% of control cells (CM) exhibited an excitatory response to pulses of ACh (n = 30, Fig. 2 a). In the presence of U0126 however, only 22% of cells exhibited an excitatory response to ACh (n = 9; Fig. 2 aii), similarly to cells that had been plated in the absence of CM (DM; n = 8; [χ^2^ (1) = 2.015, *P* = 0.156]) (Fig. 2 b). It thus appears that ERK1/2 activity is required for CM-induced excitatory nAChR expression in single, postsynaptic LPeD1 neurons (χ^2^ (1) = 6.532, *P* = 0.011) (Fig. 2 b), demonstrating the importance of the MAPK/ERK cascade in the development of the cholinergic system in the CNS.

### Activation of MAPK/ERK is required for excitatory synapse formation

**Figure 3:**
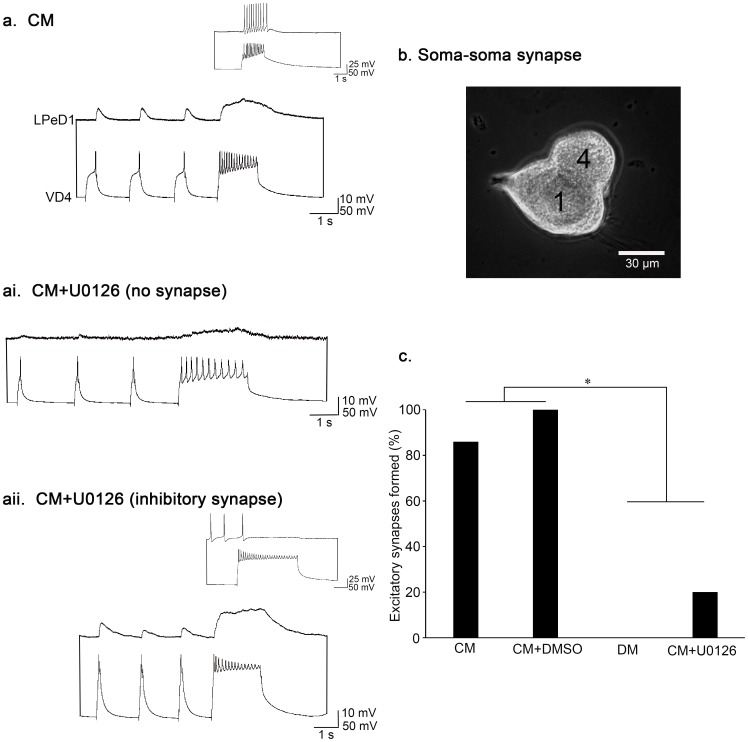
The MAP/ERK cascade is required for excitatory synapse formation. (**a**) Individual postsynaptic LPeD1 neurons were paired overnight in CM, next to the presynaptic cell, VD4. Both cells were impaled with sharp intracellular electrodes the next day, and held at −100 mV for consistency. Action potentials triggered in VD4 induced 1-for-1 EPSPs in LPeD1 (n = 29). Inset: A train of action potentials triggered in VD4 induced spiking in LPeD1 when held at −60 mV, confirming the presence of an excitatory synapse. (**ai–aii**) In the presence of the MEK1/2 inhibitor, the incidence of excitatory synapse formation was reduced (n = 20). In most cases, either no synapses formed (ai), or they were inhibitory (aii). (**aii**) The PSPs shown are reversed IPSPs. Inset: A train of action potentials triggered in VD4 induced a cessation of firing in LPeD1 when held at −60 mV, confirming the presence of an inhibitory synapse. (**b**). Experimental preparation. Phase contrast image of a soma-soma synapse imaged at 20× magnification. 4 = VD4. 1 = LPeD1. (**c**) Summary of the percentage of pairs that formed an excitatory synapse with a significance of *P*<0.001, as determined using Pearson's Chi-squared test.

We next sought to determine whether the MAPK/ERK cascade was required for excitatory synapse formation between pairs of neurons. Individual LPeD1 cells were plated next to single VD4 neurons in a soma-soma configuration (see Fig. 3 b) in CM, CM+DMSO (vehicle control; 0.4%), CM+U0126 (40 µM), or DM. After 14–18 hours of pairing, we recorded from both VD4 and LPeD1 intracellularly, to determine if an excitatory synapse had formed. In CM, 86% of pairs formed an excitatory synapse (n = 29; Fig. 3 a). Similar to CM, 100% of pairs formed excitatory synapses when plated in CM with DMSO (n = 9; [χ^2^ (1) = 1.387, *P* = 0.239]). In the absence of NTFs (DM; n = 12; [χ^2^ (1) = 21.000, *P*<0.001]) (Fig. 3 c) however, none of the pairs formed an excitatory synapse. Lastly, when cells were paired in CM in the presence of the MEK inhibitor U0126, the incidence of excitatory synapse formation was significantly reduced to 20% (n = 20; [χ^2^ (1) = 21.478, *P*<0.001]) (Fig. 3c). Furthermore, the overall frequency of synapse formation (independent of synapse type) was reduced in the presence of U0126 (55%; n = 20; Fig. 3 ai) when compared to all other culture conditions (χ^2^ (3) = 18.416, *P*<0.001). In comparison, 96% of VD4-LPED1 pairs formed synapses in CM (n = 29), 100% in CM+DMSO (n = 9), and 92% in DM (n = 12). As such, we conclude that MAPK/ERK activity is necessary for synapse formation in general. More specifically, ERK is also required for CM-mediated excitatory synapse formation, by inducing the expression of excitatory nAChRs in the postsynaptic neuron.

### CM-derived trophic factors upregulate the expression of *L-MEN1* mRNA

**Figure 4:**
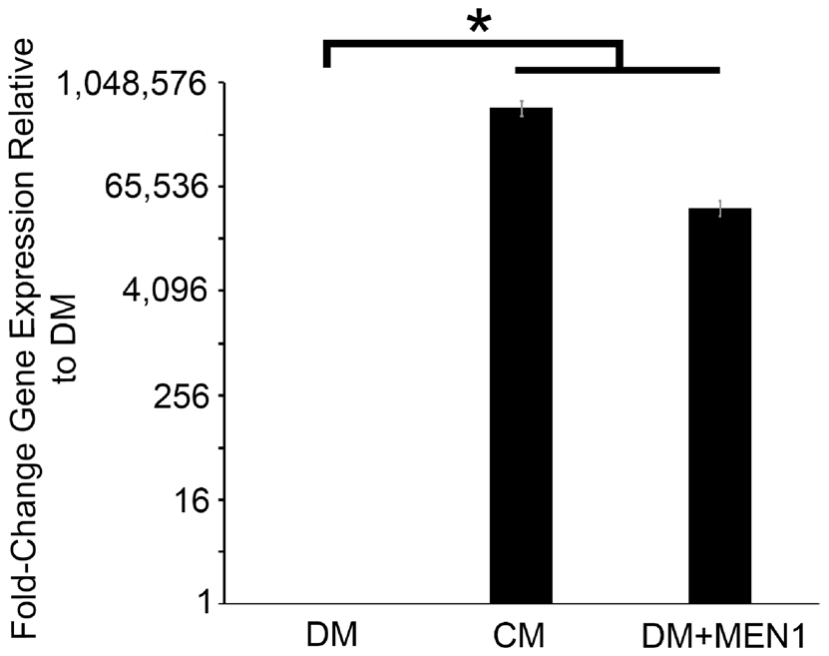
Menin expression is upregulated by neurotrophic factors, and following microinjections of synthetic *L-MEN1* mRNA. Summary of the relative gene expression of *L-MEN1*, quantified via qPCR, with a significance of *P*(H1)<0.001 as determined using the REST randomization test (n = 3 for all conditions).

In order for NTFs to induce the expression of excitatory nAChRs in LPeD1 neurons, we predicted that there was a transcription factor targeted downstream of RTK activation. As already mentioned, we have shown previously that the tumor suppressor, *L-MEN1*, is required for synapse formation in *Lymnaea stagnalis* [32]. Since *MEN1* can bind dsDNA [33], acting alone or in complexes to regulate transcription [34], we hypothesized that the expression of *L-MEN1* was upregulated in single LPeD1 neurons exposed to CM. To investigate this further, we extracted the cytoplasm from 12–15 LPeD1 neurons that had been plated in either CM or DM overnight using an autoclaved patch pipette tip (the nuclei were left behind in the dish to avoid contamination of the samples with genomic DNA). Next, cDNA was synthesized via RT-PCR, followed by amplification of *L-MEN1* using gene-specific primers (Table 1). After pooling the samples, relative gene expression was quantified via qPCR on 3 separate plates (n = 3), each run in triplicate (see Table 2 for primers used). The expression of *L-MEN1* in CM was significantly upregulated more than 500, 000 fold compared to DM (n = 3; Fig. 4; REST randomization test, *P*(H1)<0.001), demonstrating that the expression of the novel synaptogenic molecule, *L-MEN1*, is regulated by NTFs in LPeD1 neurons.

### Injection of synthetic *L-MEN1* mRNA into single neurons is sufficient to induce the expression of excitatory nAChRs

We hypothesized that expression of *L-MEN1* is sufficient to induce the expression of excitatory nAChRs in the absence of NTFs. To test our hypothesis, we cultured single LPeD1 neurons in CM or DM. After allowing the neurons to settle for at least 15 min, we microinjected synthetic *L-MEN1* mRNA into the cell bodies of neurons plated in DM, using a sterile, low resistance glass microelectrode and incubated them over night. The next day (16–18 hours later), the neurons were recorded from intracellularly, while pulses of ACh (10^−6^ M) were pressure-applied to their somata, to determine if an excitatory (appropriate) or inhibitory (inappropriate or ‘default’) electrical response was present.

**Figure 5:**
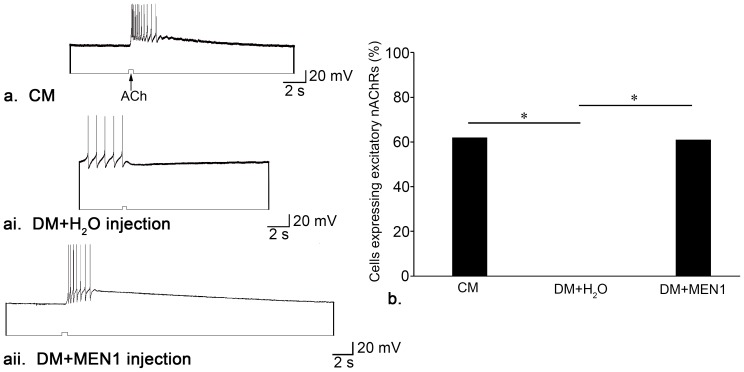
*L-MEN1* is sufficient to induce excitatory nAChR expression in the absence of neurotrophic factors. (**a**) Single LPeD1 neurons were cultured in CM and impaled with intracellular electrodes. Exogenous application of ACh (10^−6^ M) triggered an excitatory response in the postsynaptic neuron, LPeD1 (n = 34). (**ai**) In the absence of neurotrophic factors, inappropriate inhibitory nAChRs were expressed by ‘default’ (n = 15). (**aii**). Microinjections of synthetic *L-MEN1* rescued excitatory nAChR expression in the absence of CM (n = 33). (**b**) Summary of the percentage of cells that functionally exhibited excitatory nAChRs with a significance of *P*<0.001, as determined using Pearson's Chi-squared test.

We first established that *L-MEN1* mRNA was present in LPeD1 neurons following microinjection. Single cell qPCR was used to determine that there was a significant increase in *L-MEN1* mRNA abundance by more than 35, 000 fold following microinjection of synthetic *L-MEN1* mRNA (n = 3; Fig. 4; REST randomization test, *P*(H1)<0.001). When plated in CM, 62% of the cells were excited by ACh (n = 34; Fig. 5 a). In contrast, neurons plated in DM (and injected with water), did not express excitatory nAChRs (n = 15; [χ^2^ (1) = 16.213, *P*<0.001]) (Fig. 5 ai). Similar to CM, 61% of the cells that were cultured in DM and microinjected with *L-MEN1* expressed excitatory nAChRs (n = 33; [χ^2^ (1) = 0.009, *P* = 0.922]) (Fig. 5 aii), a significantly higher incidence of expression than that exhibited by cells injected with water only (n = 15; [χ^2^ (1) = 15.584, *P*<0.001]) (Fig. 5b). We have thus discovered a novel function for menin in the CNS of *Lymnaea*, and report that L-menin is sufficient to induce the expression of excitatory nAChRs in postsynaptic LPeD1 neurons, independent of presynaptic signalling.

### Injection of synthetic *L-MEN1* mRNA into single postsynaptic neurons is sufficient to induce excitatory synapse formation

We next sought to determine whether L-menin was also sufficient to induce excitatory synapse formation between paired neurons in the absence of NTFs. To this end, we cultured single LPeD1 neurons in either DM or CM. After allowing the neurons to adhere to the dishes in DM for at least 15 min, we microinjected their somata with water (negative control) or synthetic *L-MEN1* mRNA. Following injection, single VD4s were placed next to the LPeD1 neurons, in a soma-soma configuration (Fig. 3 b) and left overnight (14–18 hrs). The next day, we recorded from each neuron intracellularly to determine if an excitatory synapse had formed.

**Figure 6:**
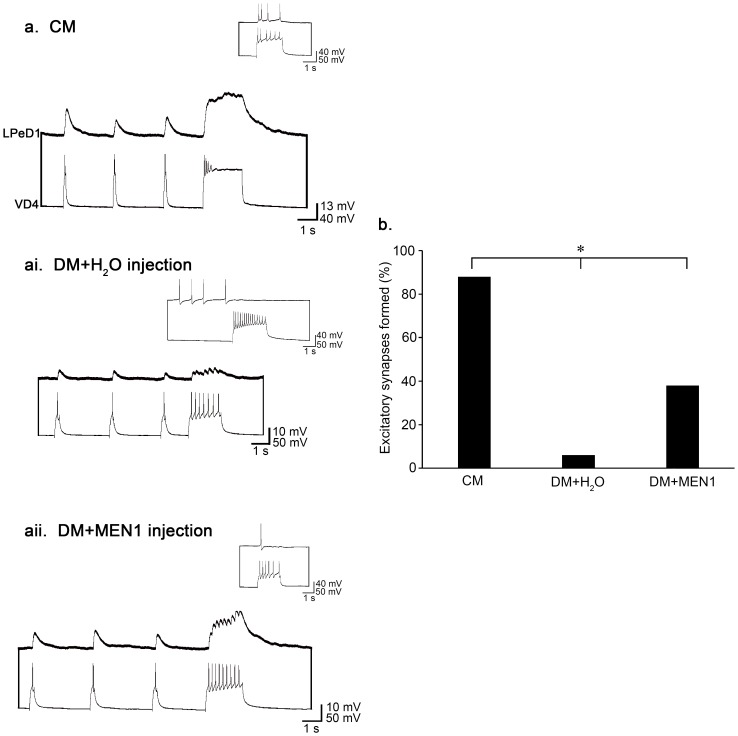
*L-MEN1* is sufficient to induce excitatory synapse formation in the absence of neurotrophic factors. (**a**) Individual postsynaptic LPeD1 neurons were paired next to the presynaptic cell, VD4, overnight in CM. Both cells were impaled with sharp intracellular electrodes the next day, and held at −100 mV for consistency. Action potentials triggered in VD4 induced 1-for-1 EPSPs in LPeD1 (n = 17). Inset: A train of action potentials triggered in VD4 induced spiking in LPeD1 when held at −60 mV, confirming the presence of an excitatory synapse. (**ai**). In the absence of neurotrophic factors, inappropriate inhibitory synapses formed by ‘default’ (n = 17). The PSPs shown are reversed IPSPs. Inset: A train of action potentials triggered in VD4 induced a cessation of firing in LPeD1 when held at −60 mV, confirming the presence of an inhibitory synapse. (**aii**). Microinjections of synthetic *L-MEN1* rescued excitatory synapse formation in the absence of CM (n = 16). Inset: LPeD1 was held at −60 mV to confirm that the synapses formed were excitatory. (**b**) Summary of the percentage of pairs that formed an excitatory synapse with a significance of *P*<0.05, as determined using Pearson's Chi-squared test.

The overall frequency of synapse formation was not affected by the exogenous expression of *L-MEN1*(χ^2^ (2) = 2.609, *P* = 0.271). Only the synapse type (whether the synapses formed were excitatory in inhibitory) was affected by the presence or absence of trophic factors or *L-MEN1*. When cultured in CM, 94% of VD4-LPeD1 pairs formed synapses (n = 17). When cultured in DM, 89% of cell pairs formed synapses when injected with water (n = 17), and 75% of cell pairs formed synapses when injected with *L-MEN1* (n = 16). When cultured in CM, 88% of cell pairs formed an excitatory synapse (Fig. 6 a). In contrast, only 6% of the water-injected DM pairs formed an excitatory synapse, significantly less than observed in CM ([χ^2^ (1) = 23.139, *P*<0.001]; Fig. 6 ai). However, when LPeD1 was injected with *L-MEN1*, 38% of pairs cultured in DM formed excitatory synapses (Fig. 6 aii), which was significantly higher than those pairs in which LPeD1 had been injected with water alone [χ^2^ (1) = 4.930, *P* = 0.026]. Nevertheless, the incidence of excitatory synapse formation was still higher in CM than the *L-MEN1*-injected pairs cultured in DM [χ^2^ (1) = 9.169, *P* = 0.002] (Fig. 6 b), suggesting that the exogenous expression of L-menin is either not able to completely rescue synapse formation in the absence of NTFs, or is not being successfully expressed in every pair.

Finally, we compared the strength of excitatory synapses formed in CM to those that were successfully rescued in DM by *L-MEN1* microinjections. In order to quantify synaptic strength, we measured the amplitudes of excitatory postsynaptic potentials (EPSPs) in LPeD1, which were induced by action potentials triggered in VD4. All LPeD1 neurons were held at −100 mV for consistency. We found that there was no significant difference between the synaptic strength of excitatory synapses formed in CM and those that had been formed in DM and rescued by *L-MEN1* injection (t-test; *P* = 0.523). Specifically, synapses formed in CM exhibited a mean EPSP amplitude of 10.4±1.2 mV, whereas those formed in DM that expressed exogenous L-menin were 7.5±1.9 mV in amplitude. Therefore, when synapse formation was successfully rescued by *L-MEN1* microinjection, the synapses appeared to be as strong, or mature, as those having formed in the presence of NTFs.

### *L-MEN1* microinjections rescue the expression of excitatory nAChRs *in vitro*

We next hypothesized that L-menin could rescue deficits in nAChR expression caused by the inhibition of MAPK/ERK cascades and L-type VGCCs. To test this hypothesis, single LPeD1 neurons were cultured in DM. The cells were then incubated in either 40 µM U0126 for 2 hours, 10 µM nifedipine for 1 hour, or the appropriate vehicle control (0.4% DMSO for U0126 experiments, and 0.1% DMSO for nifedipine experiments). Following drug incubation, the neurons were injected with either synthetic *L-MEN1* or molecular-grade water. The media (DM) was then replaced with fresh media (CM) and the appropriate concentration of U0126 or nifedipine was added. The next day (16–18 hours later), the neurons were recorded from intracellularly, while pulses of ACh (10^−6^ M) were pressure-applied to their somata, to determine if an excitatory (appropriate) or inhibitory (inappropriate or ‘default’) electrical response was present.

**Figure 7:**
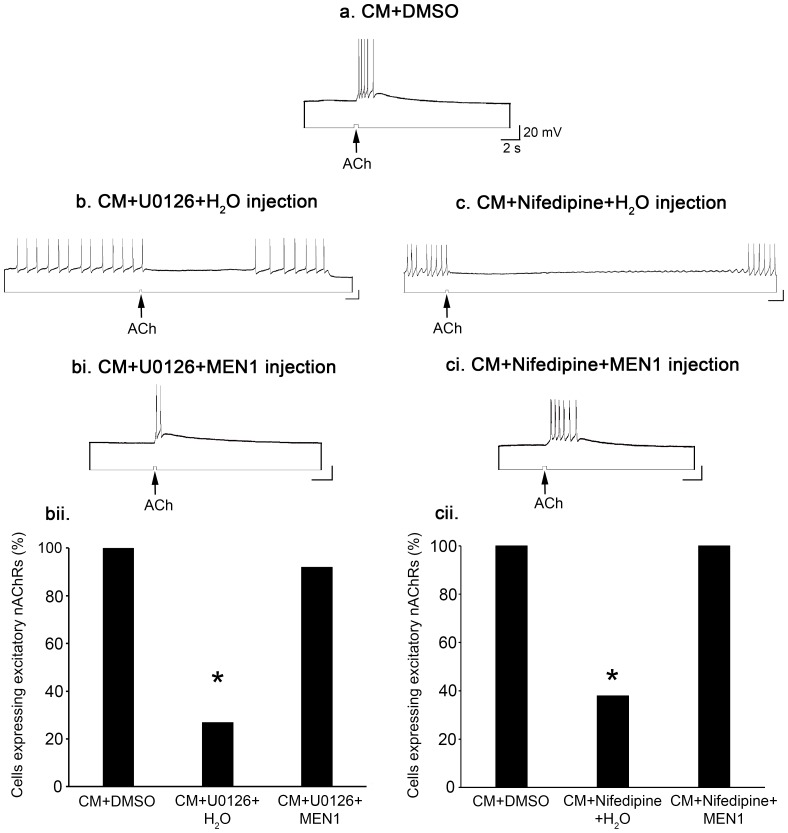
Microinjections of *L-MEN1* rescue excitatory nAChR expression *in vitro*. All vertical (voltage) scale bars represent 20 mV. All horizontal (time) scale bars represent 2 s. (**a**) Single, postsynaptic LPeD1 neurons were cultured in CM+DMSO (vehicle control; n = 8 for U0126 experiment; n = 16 for nifedipine experiment) and impaled with intracellular electrodes. Exogenous application of ACh (10^−6^ M) triggered an excitatory response. (**b**) Exogenous application of ACh to LPeD1 somata induced an inhibitory response in the presence the MEK1/2 inhibitor U0126 (n = 11) (40 µM). (**bi**) The deficit in excitatory nAChR expression due to inhibition of the MAPK/ERK cascade in LPeD1 neurons was rescued by microinjections of synthetic *L-MEN1* (n = 12). (**bii**) Summary of the percentage of cells that functionally exhibited excitatory nAChRs with a significance of *P*<0.01, as determined using Pearson's Chi-squared test. (**c**) LPeD1 neurons expressed inappropriate inhibitory nAChRs when cultured in the L-type VGCC blocker nifedipine (n = 13) (10 µM). (**ci**) Microinjections of synthetic *L-MEN1* rescued excitatory nAChR expression in LPeD1 neurons cultured in nifedipine (n = 12). (**cii**) Summary of the percentage of cells that functionally exhibited excitatory nAChRs with a significance of *P*<0.001, as determined using Pearson's Chi-squared test.

Microinjections of *L-MEN1* rescued the expression of functional excitatory nAChRs in postsynaptic neurons treated with the MEK inhibitor U0126 ([χ^2^ (4) = 16.496, *P* = 0.002]; Fig. 7 b–bii), as well as the L-type VGCC inhibitor nifedipine ([χ^2^ (4) = 28.210, *P*<0.001]; Fig. 7 c–cii). When cells were injected with water alone, only 27% of LPeD1 neurons expressed excitatory nAChRs when exposed to U0126 (n = 11), and 38% when cultured in nifedipine (n = 13). On the other hand, when the cells were injected with synthetic *L-MEN1*, 92% expressed functional excitatory nAChRs in media containing U0126 (n = 12), and 100% in media containing nifedipine (n = 9). This suggests that exogenous expression of L-menin is sufficient to induce the expression of excitatory nAChRs, even in the presence of MAPK/ERK and L-type VGCC inhibitors.

### L-EGFR is required for synapse formation *in situ*

**Figure 8:**
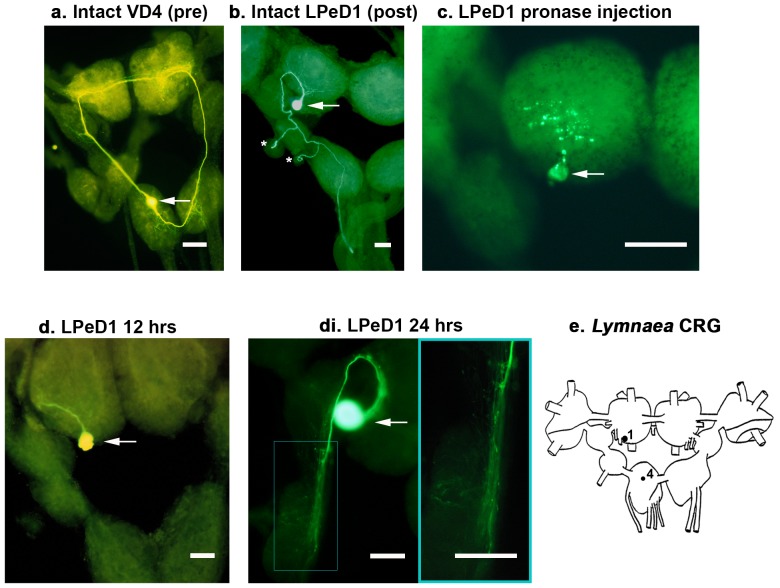
Lucifer yellow images illustrating the single cell transplantation procedure. Arrows point to the somata of intact or transplanted neurons. Scale bars represent 100 µm. (**a**) The presynaptic neuron, VD4, is located in the visceral ganglia, and is characterized by two axons that span the entire central ring ganglia (CRG; 4× magnification). (**b**) The postsynaptic neuron, LPeD1, is located in the pedal ganglia, and has one axon which extends downwards toward the visceral ganglia, where it makes a synaptic connection with VD4 as well as many other neurons. In addition, LPeD1 projects axonal branches through peripheral nerves to innervate various organs (marked by asterisks; 4× magnification). (**c**) The native LPeD1 neuron is first ablated in the host ganglia via pronase injection (5% solution in *Lymnaea* saline) and is completely fragmented within 1 hr (10× magnification). (**d**) An LPeD1 neuron from a donor animal is then transplanted into the original location of the ablated LPeD1 in the host CRG, where it exhibits significant regeneration within 12 hours (4× magnification). (**di**) Regeneration 24 hours post-transplantation (10× magnification). Inset shows magnified details of neurite outgrowth extending down through the left parietal and pleural ganglia. (**e**) Schematic of the *Lymnaea* CRG, indicating the relative size and position of LPeD1 (1) and VD4 (4).

We next sought to decipher whether the endogenous NTFs that promote synapse formation *in vitro*, also function through the L-EGFR *in situ*. Our first hypothesis was that knock-down of the L-EGFR would perturb synapse formation in intact *Lymnaea* CRG (Fig. 8 e). To investigate this, individual LPeD1 neurons were isolated from the CRG, and incubated for 24 hours in either DM (control), *Lymnaea* acetylcholine binding protein dsRNA (L-AChBP; glial protein; negative control; 200 ng/ml in DM), or L-EGFR dsRNA (200 ng/ml in DM). Following these treatments, the neurons were then implanted into a fresh CRG, in which the host LPeD1 had been previously ablated by pronase injection (5% solution in *Lymnaea* saline), and allowed to regenerate (Fig. 8 c–di). Synapse formation was tested 48–72 hours post-transplantation via intracellular recording.

**Figure 9:**
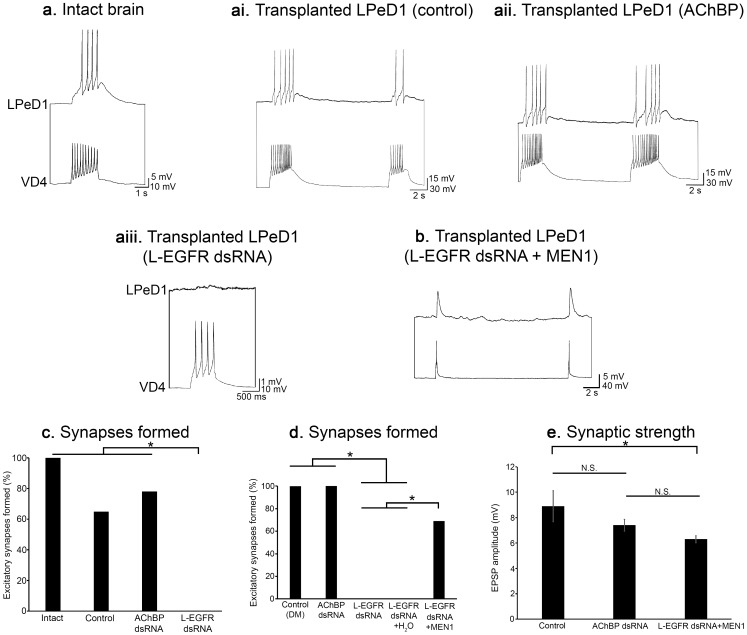
Synthetic *L-MEN1* rescues deficits in synapse formation induced by L-EGFR knockdown *in situ*. (**a**) Intact VD4 and LPeD1 neurons were simultaneously impaled with intracellular electrodes to confirm whether or not a synapse was present. Trains of action potentials triggered in VD4 induced a corresponding excitatory response in LPeD1 (n = 7). (**ai**) Control, LPeD1 transplants re-formed excitatory, cholinergic synapses with intact, host VD4 neurons (n = 17). (**aii**) LPeD1 neurons pre-treated with L-AChBP dsRNA (negative control; 200 ng/ml) prior to transplantation established excitatory synapses with host VD4 neurons (n = 9). (**aiii**) Incubation of LPeD1 neurons with L-EGFR dsRNA (200 ng/ml) prior to transplantation prevented excitatory synapse formation with host VD4 neurons (n = 17). (**b**) Whole CRG were pre-treated with L-EGFR dsRNA prior to isolation of the LPeD1 transplants. 4–6 hours later, LPeD1 was injected with *L-MEN1* mRNA. 2–3 hours following injection, the LPeD1 neurons were isolated and transplanted into host CRG, where they re-formed excitatory synapses with VD4 (n = 13). (**c–d**) Summary of the percentage of pairs that formed an excitatory synapse with a significance of *P*<0.01, as determined using Pearson's Chi-squared test. (**e**) Summary of mean EPSP amplitude (mV) with a significance of *P*<0.05, as determined using a univariate ANOVA with Tukey's *post hoc* test. Error bars represent standard error of the mean (SEM).

We first confirmed that excitatory synapses were present between VD4 and LPeD1 *in situ*, in CRG that had not been perturbed. In 100% of preparations, the intact neurons formed an excitatory synapse (n = 7; Fig. 9 a). Similarly, 65% of control (DM incubated) LPeD1 neurons transplanted into host CRG, re-formed excitatory, cholinergic synapses with VD4 (n = 17; Fig. 9 ai; [χ^2^ (1) = 3.294; *P* = 0.07]). In 78% of cases, excitatory synapses also formed between transplanted LPeD1 neurons incubated in L-AChBP dsRNA and host VD4 (n = 9; Fig. 9 aii), similar to both intact CRG [χ^2^ (1) = 1.778, *P* = 0.182], and control, DM-incubated LPeD1 transplants [χ^2^ (1) = 0.472, *P* = 0.492]. Even though neurite outgrowth was unperturbed, 0% of LPeD1 transplants pre-treated with L-EGFR dsRNA re-formed excitatory synapses *in situ* (n = 17; Fig. 9 aiii), which was significantly different from intact CRG [χ^2^ (1) = 24.000, *P*<0.001], DM-treated transplants [χ^2^ (1) = 16.261, *P*<0.001], and L-AChBP-treated transplants [χ^2^ (1) = 18.094, *P*<0.001]. We have thus concluded that: (1) the NTFs promoting synapse formation *in situ*, function through the L-EGFR, (2) the synaptogenic actions of L-EGFR signalling are independent of theirs role(s) in regeneration (neurite outgrowth was unaffected by L-EGFR knock-down), and (3) activation of the L-EGFR is required for the expression of nAChRs *in situ* (data not shown) (Fig. 9 c).

### *L-MEN1* microinjections rescue synapse formation *in situ*

We next asked whether the deficit in synaptogenesis induced by L-EGFR knockdown could be rescued by *L-MEN1* microinjection. To test this possibility, we incubated whole CRG for 4–6 hours in either DM alone (control), L-AChBP dsRNA (negative control; 200 ng/ml), or L-EGFR dsRNA (200 ng/ml). Two other groups of CRG incubated in L-EGFR dsRNA were also included, in which half received H_2_O injections into LPeD1 (injection control), and the other half were injected with synthetic *L-MEN1* mRNA into LPeD1. Following treatment, LPeD1 neurons were isolated and transplanted into a fresh host ganglia, in which the native LPeD1 had been previously ablated by pronase injection (5% in *Lymnaea* saline).

In 100% of the CRG tested, both the control LPeD1 transplants (n = 5) and the transplants incubated in L-AChBP dsRNA (n = 6) formed excitatory synapses with the host VD4 neurons. In contrast, 0% of LPeD1 transplants re-formed their excitatory, cholinergic connections within the host ganglia when previously incubated in L-EGFR dsRNA (n = 5; significantly different from both control transplants [χ^2^ (1) = 11.000, *P* = 0.001] and transplants incubated in L-AChBP dsRNA [χ^2^ (1) = 11.000, *P* = 0.001]). However, when LPeD1 transplants were injected with synthetic *L-MEN1* mRNA following L-EGFR knockdown (n = 13), synapse formation was rescued (Fig. 9 b). In such cases, 69% of transplants re-formed excitatory connections with host VD4, significantly more than in those preparations in which LPeD1 had been injected with H_2_O alone (0%; n = 5; [χ^2^ (1) = 6.923, *P* = 0.009]). The incidence of synapse formation between LPeD1 transplants injected with *L-MEN1* and host VD4, was not different from control transplants [χ^2^ (1) = 1.978, *P* = 0.160] (Fig. 9 d).

Finally, we measured the synaptic strength of the synapses rescued by injection of synthetic *L-MEN1* mRNA, and compared it to the strength of excitatory synapses formed in control conditions (all LPeD1 neurons were held at −100 mV for consistency). A univariate ANOVA revealed a significant effect of treatment on EPSP amplitude [F_2,16_ = 4.712; *P* = 0.025]. The mean amplitude of EPSPs between control LPeD1 transplants and host VD4 neurons was 8.9±1.2 mV, which was not significantly different from those synapses formed between LPeD1 transplants incubated in L-AChBP dsRNA and host VD4 neurons (7.4±0.5 mV; *P* = 0.296; Tukey's *post hoc* test). While the strength of synapses rescued by *L-MEN1* injection (6.3±0.3 mV) was not significantly different from the L-AChBP knock-down group (*P* = 0.412; Tukey's *post hoc* test), it was significantly different from the control transplant group (*P* = 0.019; Tukey's *post hoc* test), demonstrating that while L-menin is capable of rescuing synapse formation *in situ*, the synapses may not be as strong as those formed in control conditions (Fig. 9 e). This may suggest that other additional synaptogenic factors may contribute to the maturation of synapses formed in *Lymnaea* CRG. Nonetheless, we have demonstrated, for the first time, that postsynaptic L-menin is sufficient to induce synapse formation in the intact CNS.

## Discussion

It is speculated that neurological disorders, ranging from autism, epilepsy to severe types of mental retardation, all depend upon proper synaptic connectivity established during development [24]. While the importance of bidirectional communication between the synapse and nucleus during neuronal development and plasticity has been recognized [27], we still have much to learn about the mechanisms mediating the initiation, maturation, and stabilization of synapses in the CNS. Moreover, as opposed to contact-mediated cell-cell signalling, the role of extrinsic molecules in synaptic development and plasticity remain largely unknown.

In this study, we have investigated the mechanisms by which NTFs such as L-EGF, signal through RTKs to induce cholinergic synapse formation between pairs of VD4 and LPeD1 neurons in *Lymnaea stagnalis*. To this end, we have uncovered a novel role for the MAPK/ERK cascade in synaptogenesis, demonstrating that it is necessary for the NTF-induced expression of excitatory nAChRs in the postsynaptic neuron LPeD1, and subsequent synapse formation between LPeD1 and VD4. Due to the fact that MEK inhibition impairs the overall frequency of synapse formation (independent of synapse type), it may also be the case that the MAPK/ERK cascade plays an as of yet undiscovered role in presynaptic development, or in the clustering of postsynaptic nAChRs at the synaptic contact site. Previously, we found that the tumor suppressor protein menin, was required in the postsynaptic cell for synapse formation in *Lymnaea* [32], demonstrating for the first time that this protein played a key role in nervous system development. We extend these findings here, identifying a novel link between NTFs and the expression of *L-MEN1* gene in single, individually identified neurons. Specifically, in the absence of NTFs, *L-MEN1* transcript is virtually absent. When LPeD1 neurons are cultured in CM however, *L-MEN1* mRNA is upregulated more than 500, 000 fold. Furthermore, microinjection of *L-MEN1* mRNA into the cell bodies of individual LPeD1 neurons was sufficient to rescue the expression of excitatory nAChRs, both in the absence of NTFs, and in the presence of MAPK/ERK and L-type VGCC inhibitors. Microinjections of *L-MEN1* also rescued synapse formation between VD4-LPeD1 neurons paired in the absence of CM. We extend these findings to investigate synaptogenesis in the intact *Lymnaea* CNS, demonstrating that single cell knock-down of the L-EGFR prior to the transplantation of individual LPeD1 neurons perturbs both excitatory responses to ACh (not shown) and synapse formation. Finally, we show unequivocally that following L-EGFR knock-down, exogenous expression of *L-MEN1* is sufficient to rescue synapse formation *in situ*. It is worth mentioning however, that *L-MEN1* was able to rescue excitatory synapse formation to a much greater extent *in situ* (69%) than *in vitro* (38%). In culture, cell pairs were plated in the absence of all NTFs, and injected with exogenous *L-MEN1*. *In situ* however, the cell pairs were exposed to L-EGFR dsRNA prior to *L-MEN1* microinjection, but other sources of NTFs could have still been present to activate other RTKs. This may suggest that there are additional factors present in the intact CNS other than L-EGF, including other NTFs, which could contribute to excitatory synapse formation. To the best of our knowledge, this is the only study whereby a single synaptogenic molecule was found sufficient for inducing postsynaptic changes leading to excitatory synapse formation between synaptic partners in both cell culture and the intact CNS. Our data specifically demonstrates that NTFs can function independently of cell-cell signalling and activity-dependent mechanisms, to prime the postsynaptic cell for synapse formation prior to contact between pre- and postsynaptic neurons. Our study is also the first to demonstrate a link between NTFs and a tumour suppressor in regulating the expression of nAChRs. Our findings thus underscore the importance of cross-talk between various, seemingly unrelated, pathways in regulating cellular function.

In addition to our findings in the CNS, Xu et al. (2012) discovered a potential role of menin in the peripheral nervous system. They found that menin was upregulated in the spinal cord following peripheral nerve injury, and contributed to neuropathic hypersensitivity in mice by potentiating synaptic plasticity in neurons of the dorsal horn [35], [36]. Together with our study, it is thus reasonable to conclude that menin plays a role in synaptic physiology in both invertebrate and vertebrate species.

Menin has been proposed to act as a scaffold or adaptor protein, in the cytoplasm and/or nucleus, coordinating the signalling of multiple menin-interacting proteins [33]. Such proteins include a wide variety of transcriptional activators (e.g., SMADS), repressors (e.g., JunD), and cell signalling proteins (e.g., TGF-β), among many others [37]–[39]. Menin, in fact, binds to thousands of genetic loci [40], [41], recruiting a multitude of protein complexes to regulate gene expression. Furthermore, menin has been shown to interact with intermediate filament proteins such as glial fibrillary acidic protein (GFAP) and vimentin [42], suggesting that the cytoskeleton may play a role in sequestering menin in the cytoplasm. Stimuli such as insulin, have also been shown to induce the cytoplasmic localization of menin where it interacts with FOXO1. Furthermore, insulin can chronically downregulate the expression of menin, an effect that appears to be post-translational and to rely on the MAPK/ERK cascade [43]. Studies in these species however, have not been able to demonstrate a putative role for menin in synapse formation.

While the molecular players coordinating synapse formation at the NMJ have been well-described [1], the synaptogenic molecules orchestrating pre- and postsynaptic differentiation in the CNS remain to be defined. Agrin, which is released from the presynaptic nerve terminal at the NMJ, signals through the RTK, muscle-specific kinase (MuSK) receptor, to govern the aggregation and stabilization of postsynaptic nAChR clusters [1]. We propose a novel model here for central synapses by which NTFs such as L-EGF, activate RTKs at the plasma membrane of postsynaptic neurons, initiating a specific subset of intracellular events within the postsynaptic cell that are independent of presynaptic signalling and activity. Specifically, intracellular MAPK/ERK cascades are activated, and the expression of *L-MEN1* mRNA is induced. Collectively, these events mediate a molecular switch in the nAChR profile, from default inhibitory to excitatory, an event which is necessary for the formation of excitatory synapses between VD4 and LPeD1 neurons in *Lymnaea stagnalis*. Signalling through RTKs, the MAPK/ERK cascade, and menin may thus offer a unique mechanism by which NTFs prime the postsynaptic neuron for synaptogenesis prior to contact with the presynaptic cell. Most importantly, we have elucidated a novel synaptogenic role for the tumour suppressor menin, suggesting that tumour growth and development may involve similar signalling cascades, albeit leading to tumour formation in one case, or synaptogenesis in the other. Understanding this interplay may thus provide therapeutic insights either into cancer biology or neurodegenerative conditions such as Alzheimer's disease [2], where nAChR expression and function are compromised.
